# High cure rates of *Mycoplasma genitalium* following empiric treatment with azithromycin alongside frequent detection of macrolide resistance in Austria

**DOI:** 10.1007/s15010-024-02261-6

**Published:** 2024-04-22

**Authors:** David Chromy, Lisa Starossek, Katharina Grabmeier-Pfistershammer, Sarah Adamek, Felix Maischack, Stefanie Sammet, Birgit Sadoghi, Georg Stary, Birgit Willinger, Wolfgang Weninger, Stefan Esser, Athanasios Makristathis, Wolfgang Michael Bauer

**Affiliations:** 1 Department of Dermatology, Medical University of Vienna, Waehringer Guertel 18-20, 1090 Vienna, Austria; 2 Department of Dermatology and Venereology, University Hospital Essen, University Duisburg-Essen, Essen, Germany; 3 Department of Dermatology and Venereology, Medical University of Graz, Graz, Austria; 4 Division of Clinical Microbiology, Department of Laboratory Medicine, Medical University of Vienna, Vienna, Austria

**Keywords:** *Mycoplasma genitalium*, Men who have sex with men, Macrolide resistance, Azithromycin

## Abstract

**Background:**

*Mycoplasma genitalium* (MG) is an emerging sexually transmitted infection, often harboring resistance-associated mutations to azithromycin (AZM). Global surveillance has been mandated to tackle the burden caused by MG, yet no data are available for Austria. Thus, we aimed to investigate the prevalence of MG, disease characteristics, and treatment outcomes at the largest Austrian HIV—and STI clinic.

**Methods:**

All MG test results at the Medical University of Vienna from 02/2019 to 03/2022 were evaluated. Azithromycin resistance testing was implemented in 03/2021.

**Results:**

Among 2671 MG tests, 199 distinct and mostly asymptomatic (68%; 135/199) MG infections were identified, affecting 10% (178/1775) of all individuals. This study included 83% (1479/1775) men, 53% (940/1775) men who have sex with men (MSM), 31% (540/1754) HIV+, and 15% (267/1775) who were using HIV pre-exposure prophylaxis (PrEP). In logistic regression analysis, ‘MSM’ (aOR 2.55 (95% CI 1.65–3.92)), ‘use of PrEP’ (aOR 2.29 (95% CI 1.58–3.32)), and ‘history of syphilis’ (aOR 1.57 (95% CI 1.01–2.24) were independent predictors for MG infections. Eighty-nine percent (178/199) received treatment: 11% (21/178) doxycycline (2 weeks), 52% (92/178) AZM (5 days), and 37% ( 65/178) moxifloxacin (7–10 days) and 60% (106/178) had follow-up data available showing negative tests in 63% (5/8), 76% (44/58) and 85% (34/40), respectively. AZM resistance analysis was available for 57% (114/199)) and detected in 68% (78/114). Resistance-guided therapy achieved a cure in 87% (53/61), yet, empiric AZM-treatment (prior to 03/2021) cleared 68% (26/38).

**Conclusions:**

*Mycoplasma genitalium* was readily detected in this Austrian observational study, affected predominantly MSM and often presented as asymptomatic disease. We observed a worryingly high prevalence of AZM resistance mutations; however, empiric AZM treatment cleared twice as many MG infections as expected.

**Supplementary Information:**

The online version contains supplementary material available at 10.1007/s15010-024-02261-6.

## Introduction

*Mycoplasma genitalium* (*MG*) is an exceptionally small bacterium without a cell wall that can manifest as sexually transmitted infection (STI) [1]. It typically causes non-gonococcal urethritis or cervicitis in women and has also been associated with complications like pelvic inflammatory disease or preterm delivery [2]. Proctitis in men who have sex with men (MSM) caused by MG has also been described [3]. Nonetheless, a substantial proportion of MG infections remain entirely asymptomatic [4, 5] and it has been estimated that up to 3% of the general population are carriers of MG [6, 7]. The prevalence of MG increases to 6–17% for individuals seeking an STI clinic and for MSM using HIV pre-exposure prophylaxis (PrEP) [8–11], and a recent report described a MG prevalence of 20% in Swiss MSM living with HIV [12].

Management of MG infections can be challenging for several reasons. Culturing of MG requires an exceptionally high effort is time consuming, and thus, culture is not realizable for clinical diagnostics or phenotypic resistance analysis [13]. The gold standard to diagnose MG infections is currently a nucleic acid amplification test (NAAT) and a linked genotypic resistance analysis. There is, however, only a very limited number of commercially available kits for MG analysis hampering the access to sufficient MG diagnostics globally [14]. Furthermore, MG is intrinsically resistant to various antibiotics, limiting the current therapeutic armamentarium to tetracyclines, macrolides, and fluoroquinolones [13, 14]. Doxycycline is significantly less efficacious in clearing MG than azithromycin [14] and, therefore, can only be considered an alternative treatment option [14]. However, using azithromycin or moxifloxacin for MG management is also increasingly problematic due to antimicrobial resistance development [15]. Several studies have reported macrolide and fluoroquinolone resistance of 78–95% and 3–36%, respectively, in selected groups of MSM [12, 16–19]. For that reason, the latest MG treatment guidelines by the Center for Disease Control and Prevention (CDC) [20], the latest revision of the British Association for Sexual Health and HIV (BASHH) guidelines [21], as well as the European STI treatment guidelines [14] have incorporated a recommendation for antimicrobial resistance testing prior to MG treatment. However, the limited availability of MG resistance testing is acknowledged [14, 20].

To overcome these challenges in managing MG, global surveillance and thorough assessment of regional antimicrobial resistance dynamics have been suggested [5]. Yet, no epidemiologic data on MG, including antimicrobial resistance, are available for Austria. We thus aimed to investigate the prevalence of MG among all individuals tested for STIs at the HIV/STI outpatient clinic of the Medical University of Vienna, analyze predictors for testing positive for MG, and assess details of antimicrobial resistance and the treatment responses.

## Patients and methods

### Study design and population

For this observational single-center study, all MG tests collected at the HIV and STI outpatient clinic at the Vienna General Hospital were systematically analyzed. The Vienna General Hospital works in conjunction with the Medical University of Vienna and is Austria´s largest tertiary care facility. Notably, it is also one of the few providers of HIV and PrEP services in Austria and open for STI testing without a referral—comparable to a ‘walk-in-clinic’ design. MG NAAT first became available at our clinic in 02/2019. Since then, all individuals screened for STIs were also tested for MG until 03/2022—at this time, the latest revision of the European guidelines on the management of MG had been published advising against screening for MG [14]. Accordingly, the study population comprised people living with HIV (PLWH; usually tested annually and/or if symptomatic), PrEP users (usually tested every 3 months and/or if symptomatic), symptomatic individuals presenting for STI workup, and asymptomatic individuals presenting for STI screening. Results on MG tests were systematically retrieved, whereas clinical data were collected from the medical history.

### Definitions

Each test for MG included a unique time point at which a single individual was tested for MG at one or more locations. The tested locations were defined at the discretion of the physician based on the reported sex practices: typically, an STI workup for MSM includes pharyngeal, urethral, and anal sampling, for all women pharyngeal, urethral, and cervical sampling and for heterosexual men urethral sampling. An individual was defined as a single person, whereas an ‘episode’ was defined as at least one positive MG test for an individual. If more than one sample tested positive from a single individual at a given time (i.e., multiple sites were infected), this was also considered a single episode. If more than one positive MG test was available for a single individual at different time points and the individuals had tested negative at the affected location in between, it was considered a new episode/reinfection.

Individuals using ‘daily’ or ‘on-demand’ PrEP at the time of sample collection were considered as PrEP users. ‘History’ of HIV post-exposure prophylaxis (PEP) was defined by previous use of antiretroviral therapy (ART) other than PrEP in the absence of HIV infection. ‘History of syphilis’ was considered in individuals with positive *Treponema pallidum* specific test plus documented previous treatment for syphilis, whereas ongoing coinfection with syphilis was based on positive serology including a non-specific *Treponema pallidum* test plus clinical documentation. Coinfection with *Neisseria gonorrhoeae* or *Chlamydia trachomatis* was defined by a positive result obtained via NAAT at the time of MG infection.

### *Mycoplasma genitalium* test

A physician or trained nursing personnel performed pharyngeal, anal, and cervical sampling using the FLOQSwabs^®^ and UTM-RT mini transport medium (Copan, Italy). For urethral sampling, neat urine was collected. The DNA extraction was performed using the BD MAX™ EXK™ DNA-1 kit (Becton Dickinson, Heidelberg, Germany) followed by a multiplex PCR utilizing the BioGX Mycoplasma-Ureaplasma—OSR for BD MAX kit (BioGX, Birmingham, AL, USA) on a BD MAX system (Becton Dickinson). Azithromycin resistance analysis became available in 03/2021 and was performed using a ResistancePlus^®^ *Mycoplasma genitalium* FleXible kit (SpeeDx, London, UK) in conjunction with the GeneXpert system (Cepheid, Sunnyvale, CA, USA).

### Treatment

Treatment was based on European guidelines applicable at the respective time of infection [22]: doxycycline 100 mg twice daily for 2 weeks, azithromycin 500 mg day one followed by 250 mg for four consecutive days, or moxifloxacin 400 mg once daily for 7–10 days. A test of cure was usually performed 3–4 weeks after therapy had been completed. If no negative test had been available following treatment or a positive test that had been collected less than 3 weeks after completion of therapy (i.e., false positivity cannot be ruled out), data on treatment outcome were considered missing.

### Statistical analysis

GraphPad Prism 9 (GraphPad Software, La Jolla, CA, USA) and IBM SPSS Statistics 28 (IBM, Armonk, NY, USA) were used to perform the statistical analyses. Continuous variables are presented as mean ± standard deviation and group comparisons were performed with independent sample Student´s t-test. Nominal variables are plotted as number and percentage of individuals with a specific feature and group comparison was done by Pearson’s Chi-squared test or Fisher’s exact test. Binary logistic regression models were applied to investigate risk factors to test positive for MG. A sub-analysis for PrEP users with consecutive follow-up was computed using a survival analysis and presented as Kaplan–Meier curves. The level of significance for the statistical analyses was set at 0.05.

### Ethics

The present study complies with the ethical standards of the 1964 Declaration of Helsinki and its later amendments. The ethical approval was provided by the respective local ethics committees, i.e., the Medical University of Vienna (2175/2020). Due to the retrospective design, the need for an informed consent had been waived.

## Results

### Prevalence of *Mycoplasma genitalium*

**Fig. 1:**
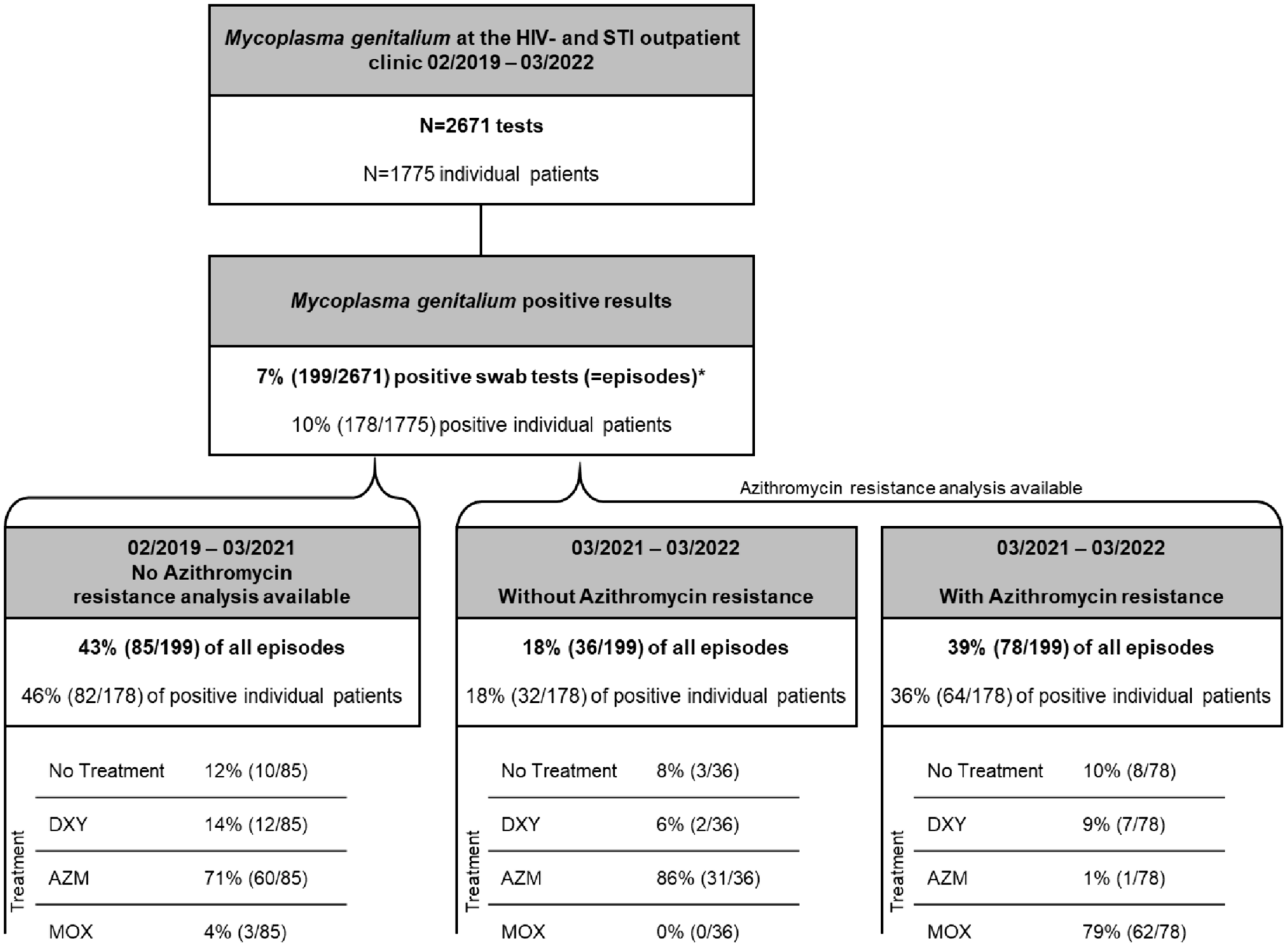
Flow chart. AZM, azithromycin; DXY, doxycycline; HIV, human immunodeficiency virus; MOX, moxifloxacin; STI, sexually transmitted infection

**Fig. 2:**
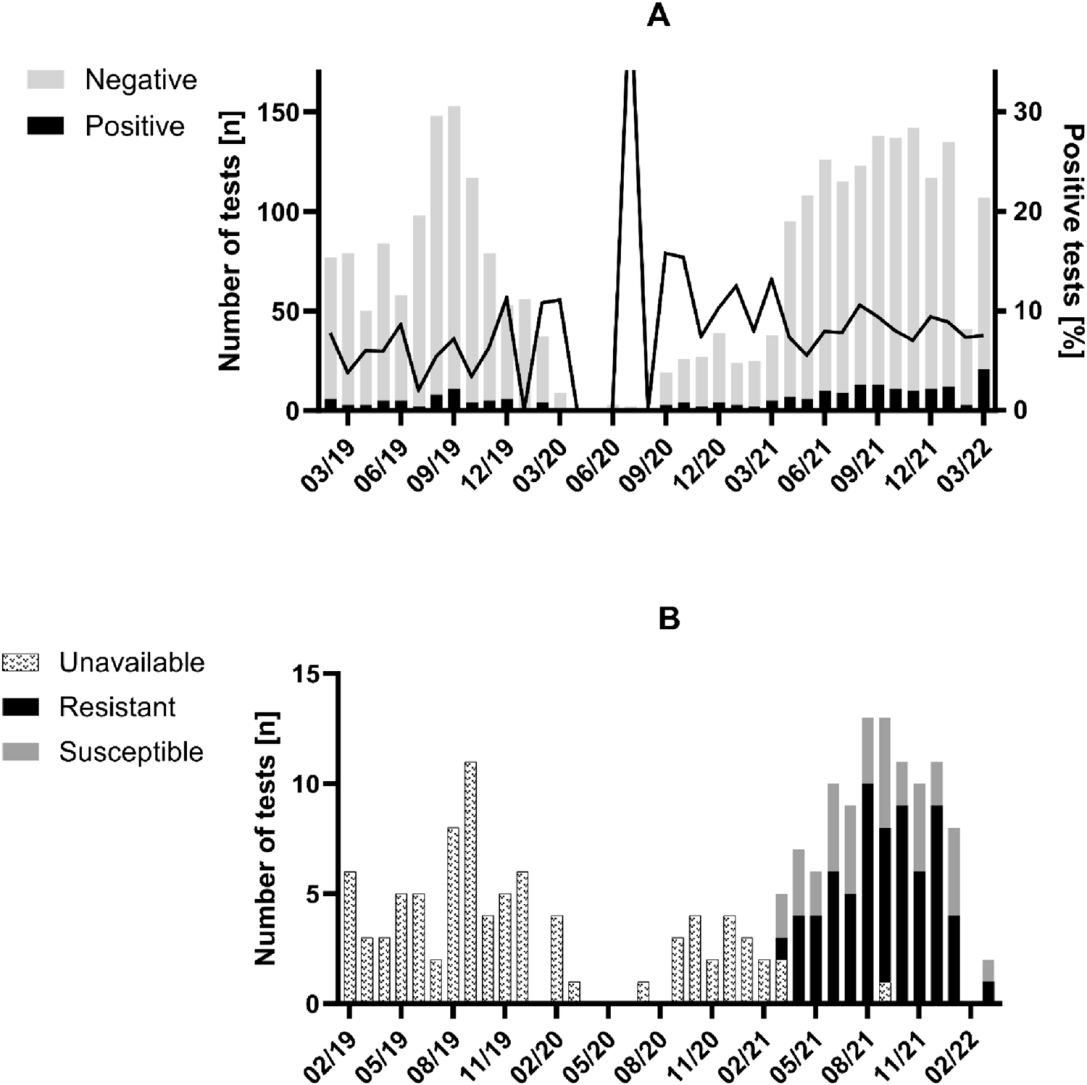
*Mycoplasma genitalium* test results per month. Each column denotes the absolute number of performed tests for *Mycoplasma genitalium* positivity (**A**) and *Mycoplasma genitalium* azithromycin resistance analysis (**B**), respectively. The line in **A** provides details on the proportion of positive tests per month. Notably, limited access to sexually transmitted infection screening followed by temporary non-availability of *Mycoplasma genitalium* PCR test kits led to a dramatic decrease in diagnosed infections from 02/2020 to 09/2020

Throughout the observational period of 3 years, 2671 tests for MG were performed in 1775 individuals. The mean age of the study population was 35.6 ± 10.7 years, the majority were male (83%, 1479/1775), and 53% (940/1775) were MSM. A substantial number of tested individuals were PLWH (30%, 540/1775) or PrEP users (15%, 267/1775) and had previously been infected with syphilis (27%, 486/1775). The overall MG prevalence was 7% (199/2671); however, 10% (178/1775) of all individuals were tested at least once positive for MG (Fig. 1). Within the first 2 years in which no azithromycin resistance testing was available, 43% (85/199) of all episodes were documented. Notably, limited STI testing access and non-availability of MG NAATs led to a marked decline in MG tests from 03/2020 to 09/2020. Yet, the positivity rate remained relatively stable throughout the observational period (Fig. 2A). Sixty-eight percent (135/199) of MG infections were asymptomatic, whereas 22% (43/199), 8% (15/199), 1% (1/199), and 3% (5/199) presented with urethritis, proctitis, pharyngitis, and cervicitis, respectively (Table 1). Since coinfections with gonorrhea (13%, 26/199), chlamydia (19%, 37/199), and syphilis (14%, 28/199) were common, we performed a sub-analysis assuming that symptoms in individuals with a coinfection were solely due to chlamydia or gonorrhea and not caused by MG (Table S1). In that ‘over-corrected’ scenario, up to 77% of MG episodes would have been considered asymptomatic.

**Table 1:** Patient characteristics and comparison of azithromycin-resistant *Mycoplasma genitalium* episodes vs. all other episodes

	All episodes *N* = 199	Azithromycin resistance negative or non-available episodes *N* = 121	Azithromycin-resistant episodes *N* = 78	*p* value
Age	35.6 ± 10.7	34.9 ± 11.7	36.9 ± 9.0	0.203
Male	93% (185/199)	89% (108/121)	99% (77/78)	0.011
MSM	81% (161/199)	73% (88/121)	94% (73/78)	<0.001
On HIV PrEP	39% (78/199)	35% (42/121)	46% (36/78)	0.106
HIV	33% (65/199)	30% (36/121)	37% (29/78)	0.275
History of HIV PEP	12% (23/199)	10% (12/121)	14% (11/78)	0.367
History of syphilis	44% (87/199)	42% (51/121)	46% (36/78)	0.578
Leading symptom				
Asymptomatic	68% (135/199)	65% (79/121)	72% (56/78)	0.004
Urethritis	22% (43/199)	27% (33/121)	13% (10/78)	
Proctitis	8% (15/199)	3% (4/121)	14% (11/78)	
Pharyngitis	1% (1/199)	1% (1/121)	0% (0/78)	
Cervicitis	3% (5/199)	3% (4/121)	1% (1/78)	
Site of manifestation^a^				
Urethral	55% (110/199)	67% (81/121)	37% (29/78)	<0.001
Anal	40% (80/199)	28% (34/121)	59% (46/78)	
Pharyngeal	2% (4/199)	2% (2/121)	3% (2/78)	
Cervical	3% (5/199)	3% (4/121)	1% (1/78)	
Treatment	89% (178/199)	89% (108/121)	90% (70/78)	0.913
Doxycycline	11% (21/178)	13% (14/108)	10% (7/70)	0.580
Negative follow-up test	63% (5/8)	57% (4/7)	100% (1/1)	–
Azithromycin	52% (92/178)	84% (91/108)	1% (1/70)	<0.001
Negative follow-up test	76% (44/58)	76% (44/58)	–	–
Moxifloxacin	37% (65/178)	3% (3/108)	89% (62/70)	<0.001
Negative follow-up test	85% (34/40)	N/A	85% (34/40)	–
Concomitant infection				
Gonorrhea	13% (26/199)	13% (16/121)	13% (10/78)	1
Chlamydia	19% (37/199)	22% (27/121)	13% (10/78)	0.093
Syphilis	14% (28/199)	11% (13/121)	19% (15/78)	0.093

*HIV* human immunodeficiency virus, *MSM* men who have sex with men, *PEP* post-exposure prophylaxis, *PrEP* pre-exposure prophylaxis

^a^ 17 individuals were tested positive at more than one location: all seventeen tested positive at the urethra plus 12 and 5 at the anal mucosa and cervix, respectively. For the purpose of this analysis, these 17 cases were assigned to the non-urethral (i.e., anal or cervical) site only

Furthermore, we analyzed predictors for testing positive for MG. By deploying a binary logistic regression model (Table 2), we identified male (odds ratio 2.51, 95% confidence interval 1.43–4.40), MSM (OR 3.67, 95% CI 2.53–5.32), use of PrEP (OR 3.52, 95% CI 2.50–4.96), and previous syphilis (OR 2.21, 95% CI 1.61–3.04) as risk factors for acquiring MG. In multivariate analysis, MSM, use of PrEP, and previous syphilis remained independent predictors for testing positive for MG at an adjusted OR of 2.55 (1.65–3.92), 2.29 (1.58–3.32), and 1.57 (1.01–2.24), respectively.

**Table 2:** Risk factors to have at least one positive *Mycoplasma genitalium* test result

	All individuals *N* = 1775	Individuals without at least one MG positive test *N* = 1597	Individuals with at least one MG positive test *N* = 178	Odds ratio (95% CI)	*p* value	Adjusted odds ratio (95% CI)	*p* value
Age							
≤24 years	13% (230/1775)	13% (205/1597)	14% (25/178)	1.00			
25–34 years	35% (617/1775)	35% (554/1597)	35% (63/178)	0.93 (0.57–1.5)	0.780		
≥35 years	52% (928/1775)	52% (838/1597)	51% (90/178)	0.88 (0.55–1.41)	0.595		
Sex							
Female	17% (296/1775)	18% (282/1597)	8% (14/178)	1.00			
Male	83% (1479/1775)	82% (1315/1597)	92% (164/178)	2.51 (1.43–4.40)	0.001	1.08 (0.57–2.06)	0.819
Transmission							
Heterosexual transmission or unknown	47% (835/1775)	50% 797(/1597)	21% (38/178)	1.00			
MSM	53% (940/1775)	50% (800/1597)	79% (140/178)	3.67 (2.53–5.32)	<0.001	2.55 (1.65–3.92)	<0.001
HIV Status							
No	70% (1214/1775)	77% (1214/1597)	65% (116/178)	1			
Yes	30% (540/1775)	23% (362/1597)	35% (62/178)	0.81 (0.58–1.12)	0.198		
Use of pre-exposure prophylaxis for HIV							
No	85% (1508/1775)	87% (1391/1597)	66% (117/178)	1			
Yes	15% (267/1775)	13% (206/1597)	34% (61/178)	3.52 (2.50–4.96)	<0.001	2.29 (1.58–3.32)	<0.001
History of syphilis							
No	73% (1289/1775)	74% (1188/1597)	57% (101/178)	1.00			
Yes	27% (486/1775)	26% (409/1597)	43% (77/178)	2.21 (1.61–3.04)	<0.001	1.57 (1.01–2.24)	0.013

*CI* confidence interval, *MG* mycoplasma genitalium, *MSM* men who have sex with men

### Antimicrobial resistance analysis

All positive MG tests since 03/2021 were subjected to antimicrobial resistance analysis, comprising 57% (114/199) of all isolates. Of those, 68% (78/114) harbored an azithromycin-resistant variant and the monthly proportion of resistant samples ranged from 50 to 82% during the observed period (Fig. 2B). By comparing azithromycin-resistant MG infections against all other MG episodes, we observed a significantly higher proportion of MSM (94%, 73/78 vs. 73%, 88/121; *p* < 0.001) among those resistant to azithromycin (Table 1). Furthermore, those with azithromycin resistance showed a higher percentage of anal infections (59%, 46/78 vs. 28%, 34/121; *p* < 0.001). In order to assess whether heterogeneity occurred between the first (without) and second (with azithromycin resistance test) half of the observational period within the study population, we compared patient characteristics of the two timespans (Table S2). MSM were more frequent (90%, 102/114 vs. 69%, 59/85) among those testing positive for MG in the second period (azithromycin resistance test available). Notably, MSM were also more often sampled in the more recent timespan (74%, 1041/1408 vs. 56%, 710/1263) and their positivity rate increased from 8% (59/710) to 10% (102/1041) while, for all non-MSM, it declined from 5% (26/553) to 3% (12/367).

### Treatment and outcomes

Eighty-nine percent (178/199) of all MG episodes received treatment. Doxycycline, azithromycin, and moxifloxacin were used in 11% (21/178), 52% (92/178), and 37% (65/178) of patients. While the proportion of individuals treated with doxycycline did not change significantly throughout the study period, azithromycin was the primary choice (80%, 60/75) before antimicrobial resistance analysis became available. Moxifloxacin was almost exclusively used if resistance to azithromycin was detected: 89% (62/70) of those patients were treated with moxifloxacin.

Cure rates, defined by a negative follow-up test, were available for 60% (106/178) of all treated individuals and were more often available for PrEP users (aOR 2.14, 95% CI 1.09–4.19) (Table S3). Overall, doxycycline achieved clearance of MG in 63% (5/8), azithromycin in 76% (44/58), and moxifloxacin in 85% (34/40), whereas empiric treatment with azithromycin during non-availability of resistance analysis cleared 68% (26/38). Eighteen patients failing initial treatment had information on a consecutive therapy available: two had failed on doxycycline, eleven on azithromycin, and five on moxifloxacin. Both doxycycline patients plus one after azithromycin received another course of azithromycin, whereas all others were re-treated with moxifloxacin. A test of cure was available for twelve individuals, with 42% (5/12) testing again positive for MG. Since all five were asymptomatic males, no further treatment was pursued.

## Discussion

Our study comprehensively evaluated 199 MG infections detected among 2671 tests taken from a representative sample of HIV/STI clinic attendees over 3 years. As the very first comprehensive epidemiologic data on MG in Austria, these results provide important insights into regional prevalence and antimicrobial resistance and fill a data gap in European MG surveillance. In our study, MG prevalence was stable at just below 10% and genotypic resistance to azithromycin was found in 68% of all tested isolates, predominantly affecting MSM. Surprisingly, we also observed high cure rates of 68% for empirically chosen treatment with azithromycin.

The prevalence of MG among individuals seeking an HIV/STI clinic observed in our study is comparable to other countries. A 2020 single-center study from an STI clinic in the UK found a MG prevalence of 11% [10] and another 2020 study conducted at a point-of-care STI testing facility in Spain observed a prevalence of 7% [23]. Of note, in both studies MSM were predominantly affected. A recently published systematic review analyzing MG in MSM reported a prevalence of 5% for the urethra and anal mucosa; however, the prevalence increased to 7% and 16%, respectively, in symptomatic individuals [24]. Accordingly, an increased prevalence of MG had been described for patients presenting with non-gonococcal urethritis: Pond M.J. and co-workers reported MG as the potential cause for urethritis in men in 17% [8]. Nonetheless, most MG infections remain silent without any symptom development. Up to 77% of the episodes of MG observed in our study were asymptomatic, corresponding to a German study that reported 71% of MG positive PrEP users as asymptomatic [11]. Due to this high number of asymptomatic MG carriers, it remained subject to debate whether screening for MG should be performed [7, 14, 24]. In particular, the increasing availability of PrEP and the consecutive surge in STI screening of highly exposed populations fueled this discussion—more tests will undoubtedly be accompanied by more diagnoses [9, 19]. In our sub-analysis of PrEP users, 28% tested positive for MG within 1 year. Ultimately, numerous STI guidelines have now recommended not to screen for MG in asymptomatic individuals [14, 20].

As far as we know, whether MG screening plus consecutive treatment could reduce the MG prevalence in selected populations (e.g., PrEP users) remains unclear. For chlamydia and gonorrhea, however, it has been calculated that in a scenario of 40% PrEP coverage for MSM at risk for HIV acquisition, quarterly STI checks could reduce both infections by 40% and 42%, respectively, within one decade [25]. Notably, this study did not include pharyngeal infection. In contrast, a study by Buyze J. and co-workers included pharyngeal infections and found only a negligible impact of frequent screening for gonorrhea on its prevalence among MSM [26]. This is in line with a systematic review from 2018 that reported no prevalence reduction of chlamydia and gonorrhea among MSM following screening implementation [27]. These results, however, cannot be directly applied to MG transmission dynamics since MG follows different epidemiologic characteristics [6, 13]. The increased proportion of asymptomatic carriers of MG would necessitate even higher screening efforts to reduce MG prevalence. In our study, positivity rates remained unchanged throughout 3 years of observation even though 89% of all MG episodes received treatment. Our work is clearly underpowered to demonstrate a longitudinal impact; still, we consider our work as a potential component for future modeling studies analyzing a ‘test and treat’ approach.

An important consideration regarding screening and treating MG is antimicrobial resistance emergence. MG is intrinsically susceptible to a very limited number of antibiotics and phenotypic resistance analysis is restricted to highly advanced research settings [13]. For the last decade, the empiric first-line treatment of MG was azithromycin; however, azithromycin was also used to treat chlamydia infections and gonorrhea [11]. The surge in STIs among MSM—to some extent facilitated by the upscale in STI screening—caused a high exposure to azithromycin and other antibiotics for this population [28], which, in turn, is associated with antimicrobial resistance development [29]. At the same time, numerous studies report increasing macrolide resistance rates of MG [4, 5, 12, 17, 18, 30, 31] and 68% of the MG isolates in our study harbored an azithromycin resistance mutation. Likewise, a recent report indicated that azithromycin-resistant *N. gonorrhoeae* in Europe rose from 2% in 2015 to 11% in 2019 among males [32]. However, the paradigm for the broad use of azithromycin in STI management is currently subject to change since doxycycline has shown a better efficacy for chlamydia in most settings [33, 34] and ceftriaxone monotherapy (i.e., not combined with azithromycin) for gonorrhea is now recommended by international guidelines [20, 35]. Of note, ceftriaxone monotherapy for gonorrhea was implemented at our clinic in 2013 and has been used exclusively since 2020 [36]. Whether these strategies combined with the recommendation to not screen for MG will lead to a reduction in azithromycin exposure and thus have a favorable impact on MG´s resistome will be subject to future studies. Until then, management of MG requires more differentiated strategies.

The American and European STI treatment guidelines now recommend a resistance-guided treatment of MG [14, 20]. Yet, they also appreciate the limited availability of reliable and commercially available kits for antimicrobial resistance analysis. Our clinic—a Central European tertiary care center—did not gain access to MG NAAT until 2019 and to azithromycin resistance testing until 2021; as of today, we have not implemented fluoroquinolone resistance analysis due to reliability concerns regarding the phenotypic/genotypic association of these tests [13] as well as limited availability of commercial kits.

The correlation of genotypic markers for phenotypic macrolide resistance is better established. Jensen J.S. and co-workers demonstrated increased minimum inhibitory concentrations for macrolides in 7 MG isolates after failing treatment with azithromycin and, correspondingly, detected mutations in the region V of the 23S rRNA gene [37]. Numerous other studies have either reported on the occurrence of these mutations or demonstrated a significantly higher prevalence of the genotypic markers among treatment failures [5, 8, 16, 18, 23, 30, 38, 39]. While these data imply a consistent sensitivity of 23S rRNA genotyping for macrolide treatment failure, it does not automatically suggest a reliable specificity of these markers. Trials assessing pretreatment genotypic macrolide resistance and not utilizing these results for treatment selection, thus providing a setting to determine specificity, typically used single-shot azithromycin [30, 37]. We observed relatively high cure rates of 68% following empiric treatment of a 5-day course of azithromycin from 02/2019 to 03/2021, although a worryingly high prevalence of 68% azithromycin resistance mutation was shown once testing became available. We did not see substantial changes in the patient population—at least no changes accounting for an effective doubling of the expected resistance—and we consider it unlikely that this high number of azithromycin resistance emerged within the last third of our 3-year observational period. Thus, we may speculate that 23S rRNA genotyping has limited specificity for treatment failure in a prolonged treatment course with azithromycin. Unfortunately, samples before 03/2021 were not stored and, thus, are unavailable to subject empirically treated MG isolates to resistance analysis to resolve this question.

We consider the high number of sampled individuals as the first strong aspect of this work. Secondly, we report the first data on the Austrian MG epidemiology. Furthermore, this study provides a comprehensive analysis of treatment and outcomes. While targeted therapy was not possible during the first half of the observational period due to the non-availability of resistance testing, it enabled us to investigate empiric treatment. The most important limitation of this study is its retrospective design, potentially introducing a variety of biases. It is the cause for incomplete outcome data (were available for 60%) which may have led to an over- or underestimation of treatment efficacy. Notably, treatment outcomes were more often available for PrEP users, while other patient characteristics were evenly distributed. Accordingly, in our study, individuals with missing treatment outcomes are more likely to belong to a population with a generally lower risk for harboring an azithromycin or fluoroquinolone-resistant MG strain. Therefore, we believe that the treatment efficacy in our dataset is more likely to be underestimated due to missing values. Finally, the retrospective design is also the reason why we cannot provide a post hoc resistance analysis of the isolates of the first study period.

In conclusion, the emergence of antimicrobial resistance in MG has necessitated epidemiologic surveillance and our work comprehensively fills a Central European gap by providing Austrian data. While most of our observations were in line with previous reports, we did see a discrepancy between empiric treatment outcomes following azithromycin and the results of the resistance analysis. Currently, a positive genotypic resistance test is obligatorily considered to cause treatment failure, yet our data suggest that future studies should further investigate the prevalence of 23S rRNA mutations among individuals successfully treated with macrolides.

## Supplementary Information

Below is the link to the electronic supplementary material.

Supplementary file1 (DOCX 94 KB)

## Data Availability

The data that support the findings of this study are available on request from the corresponding author.

## References

[1] Taylor-Robinson D, Jensen JS. *Mycoplasma genitalium*: from Chrysalis to multicolored butterfly. Clin Microbiol Rev. 2011;24:498–514. 10.1128/cmr.00006-11. 21734246 10.1128/CMR.00006-11 PMC3131060

[2] Read TRH, Murray GL, Danielewski JA, Fairley CK, Doyle M, Worthington K, et al. Symptoms, Sites, and significance of *Mycoplasma genitalium* in men who have sex with men. Emerg Infect Dis. 2019;25:719–27. 10.3201/eid2504.181258. 30882306 10.3201/eid2504.181258 PMC6433010

[3] Ong JJ, Aung E, Read TRH, Fairley CK, Garland SM, Murray G, et al. Clinical characteristics of anorectal *Mycoplasma genitalium* infection and microbial cure in men who have sex with men. Sex Transm Dis. 2018;45:522–6. 10.1097/olq.0000000000000793. 29465653 10.1097/OLQ.0000000000000793

[4] Dumke R, Ziegler T, Abbasi-Boroudjeni N, Rust M, Glaunsinger T. Prevalence of macrolide- and fluoroquinolone-resistant *Mycoplasma genitalium* strains in clinical specimens from men who have sex with men of two sexually transmitted infection practices in Berlin, Germany. J Glob Antimicrob Resist. 2019;18:118–21. 10.1016/j.jgar.2019.06.015. 31252154 10.1016/j.jgar.2019.06.015

[5] Machalek DA, Tao Y, Shilling H, Jensen JS, Unemo M, Murray G, et al. Prevalence of mutations associated with resistance to macrolides and fluoroquinolones in *Mycoplasma genitalium*: a systematic review and meta-analysis. Lancet Infect Dis. 2020;20:1302–14. 10.1016/s1473-3099(20)30154-7. 32622378 10.1016/S1473-3099(20)30154-7

[6] Andersen B, Sokolowski I, Østergaard L, Kjølseth Møller J, Olesen F, Jensen JS. *Mycoplasma genitalium*: prevalence and behavioural risk factors in the general population. Sex Transm Infect. 2007;83:237–41. 10.1136/sti.2006.022970. 17090566 10.1136/sti.2006.022970 PMC2659104

[7] Oakeshott P, Aghaizu A, Hay P, Reid F, Kerry S, Atherton H, et al. Is *Mycoplasma genitalium* in women the “New Chlamydia?” A community-based prospective cohort study. Clin Infect Dis. 2010;51:1160–6. 10.1086/656739. 20942656 10.1086/656739

[8] Pond MJ, Nori AV, Witney AA, Lopeman RC, Butcher PD, Sadiq ST. High prevalence of antibiotic-resistant *Mycoplasma genitalium* in nongonococcal urethritis: the need for routine testing and the inadequacy of current treatment options. Clin Infect Dis. 2014;58:631–7. 10.1093/cid/cit752. 24280088 10.1093/cid/cit752 PMC3922211

[9] Ong JJ, Baggaley RC, Wi TE, Tucker JD, Fu H, Smith MK, et al. Global epidemiologic characteristics of sexually transmitted infections among individuals using preexposure prophylaxis for the prevention of HIV infection: a systematic review and meta-analysis. JAMA Netw Open. 2019;2:e1917134. 10.1001/jamanetworkopen.2019.17134. 31825501 10.1001/jamanetworkopen.2019.17134 PMC6991203

[10] Broad CE, Furegato M, Harrison MA, Pond MJ, Tan N, Okala S, et al. High prevalence of coinfection of azithromycin-resistant *Mycoplasma genitalium* with other STIs: a prospective observational study of London-based symptomatic and STI-contact clinic attendees. Sex Transm Infect. 2021;97:63–8. 10.1136/sextrans-2019-054356. 32393529 10.1136/sextrans-2019-054356

[11] Jansen K, Steffen G, Potthoff A, Schuppe AK, Beer D, Jessen H, et al. STI in times of PrEP: high prevalence of chlamydia, gonorrhea, and mycoplasma at different anatomic sites in men who have sex with men in Germany. BMC Infect Dis. 2020;20:110. 10.1186/s12879-020-4831-4. 32033533 10.1186/s12879-020-4831-4 PMC7007644

[12] Ring A, Balakrishna S, Imkamp F, Burkard S, Triet F, Brunschweiler F, et al. High rates of asymptomatic *Mycoplasma genitalium* infections with high proportion of genotypic resistance to first-line macrolide treatment among men who have sex with men enrolled in the Zurich Primary HIV Infection Study. Open Forum Infect Dis. 2022;9:ofac217. 10.1093/ofid/ofac217. 35783686 10.1093/ofid/ofac217 PMC9246285

[13] Pitt R, Boampong D, Day M, Jensen JS, Cole M. Challenges of in vitro propagation and antimicrobial susceptibility testing of *Mycoplasma genitalium*. J Antimicrob Chemother. 2022;77:2901–7. 10.1093/jac/dkac281. 35979812 10.1093/jac/dkac281

[14] Jensen JS, Cusini M, Gomberg M, Moi H, Wilson J, Unemo M. 2021 European guideline on the management of *Mycoplasma genitalium* infections. J Eur Acad Dermatol Venereol. 2022;36:641–50. 10.1111/jdv.17972. 35182080 10.1111/jdv.17972

[15] Doyle M, Vodstrcil LA, Plummer EL, Aguirre I, Fairley CK, Bradshaw CS. nonquinolone options for the treatment of *Mycoplasma genitalium* in the era of increased resistance. Open Forum Infect Dis. 2020;7:ofaa291. 10.1093/ofid/ofaa291. 32782911 10.1093/ofid/ofaa291 PMC7408185

[16] Berçot B, Charreau I, Clotilde R, Delaugerre C, Chidiac C, Pialoux G, et al. High prevalence and high rate of antibiotic resistance of Mycoplasma genitalium infections in men who have sex with men. A sub-study of the ANRS ipergay PrEP trial. Clin Infect Dis. 2020;73:e2127–33. 10.1093/cid/ciaa1832. 10.1093/cid/ciaa1832 33305785

[17] Van Praet JT, Steyaert S, Vandecasteele S, Van Den Bergh B, Mahieu H, De Buyser S, et al. *Mycoplasma genitalium* acquisition and macrolide resistance after initiation of HIV pre-exposure prophylaxis in men who have sex with men. Sex Transm Infect. 2020;96:396–8. 10.1136/sextrans-2019-054335. 31896737 10.1136/sextrans-2019-054335

[18] De Baetselier I, Vuylsteke B, Reyniers T, Smet H, Van den Bossche D, Kenyon C, et al. Worryingly high prevalence of resistance-associated mutations to macrolides and fluoroquinolones in *Mycoplasma genitalium* among men who have sex with men with recurrent sexually transmitted infections. Int J STD AIDS. 2022;33:385–90. 10.1177/09564624211070704. 35094623 10.1177/09564624211070704

[19] Ahaus P, Schmidt AJ, Skaletz-Rorowski A, Uhrmacher M, Serova K, Kayser A, et al. Changes in the user profiles of HIV pre-exposure prophylaxis (PrEP) before and after PrEP reimbursement. J Infect Public Health. 2022;15:955–60. 10.1016/j.jiph.2022.07.012. 35926293 10.1016/j.jiph.2022.07.012

[20] Workowski KA, Bachmann LH, Chan PA, Johnston CM, Muzny CA, Park I, et al. Sexually transmitted infections treatment guidelines, 2021. MMWR Recomm Rep. 2021;70:1–187. 10.15585/mmwr.rr7004a1. 34292926 10.15585/mmwr.rr7004a1 PMC8344968

[21] Soni S, Horner P, Rayment M, Pinto-Sander N, Naous N, Parkhouse A, et al. British Association for Sexual Health and HIV national guideline for the management of infection with *Mycoplasma genitalium* (2018). Int J STD AIDS. 2019;30:938–50. 10.1177/0956462419825948. 31280688 10.1177/0956462419825948

[22] Jensen JS, Cusini M, Gomberg M, Moi H. 2016 European guideline on *Mycoplasma genitalium* infections. J Eur Acad Dermatol Venereol. 2016;30:1650–6. 10.1111/jdv.13849. 27505296 10.1111/jdv.13849

[23] Fernández-Huerta M, Barberá MJ, Esperalba J, Fernandez-Naval C, Vall-Mayans M, Arando M, et al. Prevalence of *Mycoplasma genitalium* and macrolide resistance among asymptomatic people visiting a point of care service for rapid STI screening: a cross-sectional study. Sex Transm Infect. 2020;96:300–5. 10.1136/sextrans-2019-054124. 31451540 10.1136/sextrans-2019-054124

[24] Latimer RL, Shilling HS, Vodstrcil LA, Machalek DA, Fairley CK, Chow EPF, et al. Prevalence of *Mycoplasma genitalium* by anatomical site in men who have sex with men: a systematic review and meta-analysis. Sex Transm Infect. 2020;96:563–70. 10.1136/sextrans-2019-054310. 32341023 10.1136/sextrans-2019-054310

[25] Jenness SM, Weiss KM, Goodreau SM, Gift T, Chesson H, Hoover KW, et al. Incidence of gonorrhea and chlamydia following human immunodeficiency virus preexposure prophylaxis among men who have sex with men: a modeling study. Clin Infect Dis. 2017;65:712–8. 10.1093/cid/cix439. 28505240 10.1093/cid/cix439 PMC5848234

[26] Buyze J, Vanden Berghe W, Hens N, Kenyon C. Current levels of gonorrhoea screening in MSM in Belgium may have little effect on prevalence: a modelling study. Epidemiol Infect. 2018;146:333–8. 10.1017/s0950268818000092. 29386078 10.1017/S0950268818000092 PMC9134539

[27] Tsoumanis A, Hens N, Kenyon CR. Is screening for chlamydia and gonorrhea in men who have sex with men associated with reduction of the prevalence of these infections? A systematic review of observational studies. Sex Transm Dis. 2018;45:615–22. 10.1097/olq.0000000000000824. 29485537 10.1097/OLQ.0000000000000824

[28] Kenyon C. We need to consider collateral damage to resistomes when we decide how frequently to screen for chlamydia/gonorrhoea in preexposure prophylaxis cohorts. AIDS. 2019;33:155–7. 10.1097/qad.0000000000002020. 30234609 10.1097/QAD.0000000000002020

[29] Bell BG, Schellevis F, Stobberingh E, Goossens H, Pringle M. A systematic review and meta-analysis of the effects of antibiotic consumption on antibiotic resistance. BMC Infect Dis. 2014;14:13. 10.1186/1471-2334-14-13. 24405683 10.1186/1471-2334-14-13 PMC3897982

[30] Bissessor M, Tabrizi SN, Twin J, Abdo H, Fairley CK, Chen MY, et al. Macrolide resistance and azithromycin failure in a *Mycoplasma genitalium*-infected cohort and response of azithromycin failures to alternative antibiotic regimens. Clin Infect Dis. 2015;60:1228–36. 10.1093/cid/ciu1162. 25537875 10.1093/cid/ciu1162

[31] Read TRH, Jensen JS, Fairley CK, Grant M, Danielewski JA, Su J, et al. Use of Pristinamycin for Macrolide-Resistant *Mycoplasma genitalium* Infection. Emerg Infect Dis. 2018;24:328–35. 10.3201/eid2402.170902. 29350154 10.3201/eid2402.170902 PMC5782881

[32] European Center for Disease Control and Prevention. Gonococcal antimicrobial susceptibility surveillance in the EU/EEA: summary of results for 2019. Stockholm: ECDC; 2021.

[33] Dukers-Muijrers N, Wolffs PFG, De Vries H, Götz HM, Heijman T, Bruisten S, et al. Treatment effectiveness of azithromycin and doxycycline in uncomplicated rectal and vaginal chlamydia trachomatis infections in women: a multicenter observational study (FemCure). Clin Infect Dis. 2019;69:1946–54. 10.1093/cid/ciz050. 30689759 10.1093/cid/ciz050 PMC6853690

[34] Lau A, Kong FYS, Fairley CK, Templeton DJ, Amin J, Phillips S, et al. Azithromycin or doxycycline for asymptomatic rectal *Chlamydia trachomatis*. N Engl J Med. 2021;384:2418–27. 10.1056/NEJMoa2031631. 34161706 10.1056/NEJMoa2031631

[35] Unemo M, Ross J, Serwin AB, Gomberg M, Cusini M, Jensen JS. 2020 European guideline for the diagnosis and treatment of gonorrhoea in adults. Int J STD AIDS. 2020. 10.1177/0956462420949126. 33121366 10.1177/0956462420949126

[36] Geusau A, Chromy D, Heissenberger D, Lippert K, Eder C, Heger F, et al. Resistance profiles of Neisseria gonorrhoeae isolates in Vienna, Austria: a phenotypic and genetic characterization from 2013 to 2020. Int J Antimicrob Agents. 2022;60: 106656. 10.1016/j.ijantimicag.2022.106656. 35988663 10.1016/j.ijantimicag.2022.106656

[37] Jensen JS, Bradshaw CS, Tabrizi SN, Fairley CK, Hamasuna R. Azithromycin treatment failure in *Mycoplasma genitalium*-positive patients with nongonococcal urethritis is associated with induced macrolide resistance. Clin Infect Dis. 2008;47:1546–53. 10.1086/593188. 18990060 10.1086/593188

[38] De Baetselier I, Kenyon C, Vanden Berghe W, Smet H, Wouters K, Van den Bossche D, et al. An alarming high prevalence of resistance-associated mutations to macrolides and fluoroquinolones in *Mycoplasma genitalium* in Belgium: results from samples collected between 2015 and 2018. Sex Transm Infect. 2020;97:297–303. 10.1136/sextrans-2020-054511. 32769204 10.1136/sextrans-2020-054511

[39] Nijhuis RH, Severs TT, Van der Vegt DS, Van Zwet AA, Kusters JG. High levels of macrolide resistance-associated mutations in *Mycoplasma genitalium* warrant antibiotic susceptibility-guided treatment. J Antimicrob Chemother. 2015;70:2515–8. 10.1093/jac/dkv136. 25995292 10.1093/jac/dkv136

