# Supplementary material for: High cure rates of Mycoplasma genitalium following empiric treatment with azithromycin alongside frequent detection of macrolide resistance in Austria
Source: Infection. 2024 Apr 22;52(5):1921–30. doi: 10.1007/s15010-024-02261-6 (PMC11499437; doi:10.1007/s15010-024-02261-6)
Supplement: Supplementary file 1 — Supplementary file1 (DOCX 94 KB) [file 15010_2024_2261_MOESM1_ESM.docx]

**Table-S1. Patient characteristics and comparison of azithromycin resistant Mycoplasma genitalium episodes vs. all other episodes – ‘overcorrection’ for symptoms caused by coinfections.**

|  | All Episodes  N=199 | Azithromycin resistance negative or non-available episodes  N=121 | Azithromycin resistant episodes  N=78 | p-value |
| --- | --- | --- | --- | --- |
| **Leading Symptom§** |  |  |  |  |
| Asymptomatic | 77% (153/199) | 78% (94/121) | 76% (59/78) | 0.049 |
| Urethritis | 16% (32/199) | 18% (22/121) | 13% (10/78) |  |
| Proctitis | 5% (10/199) | 2% (2/121) | 10% (8/78) |  |
| Pharyngitis | 1% (1/199) | 1% (1/121) | 0% (0/78) |  |
| Cervicitis | 2% (3/199) | 2% (2/121) | 1% (1/78) |  |

§ for this analysis it was assumed that the symptoms of all individuals with an active chlamydia or gonorrhea coinfection detected at the same sampling site as the *mycoplasma genitalium* infection were solely caused by chlamydia or gonorrhea. Thus, coinfections were categorized as asymptomatic *mycoplasma genitalium* infections.

**Table-S2. Patient characteristics of individuals with azithromycin susceptible *Mycoplasma genitalium* infections next to a comparison of patient characteristics before and after availability of azithromycin resistance analysis.**

|  | Azithromycin resistance negative  N=36 |  | Resistance analysis unavailable  N=85 | Resistance analysis available  N=114 | p-value |
| --- | --- | --- | --- | --- | --- |
| Age | 36.1 ± 11.8 |  | 34.3 ± 11.7 | 36.6 ± 9.9 | 0.142 |
| Male | 89% (32/36) |  | 89% (76/85) | 96% (109/114) | 0.091 |
| MSM | 81% (29/36) |  | 69% (59/85) | 90% (102/114) | <0.001 |
| On HIV-PrEP | 33% (12/36) |  | 35% (30/85) | 42% (48/114) | 0.330 |
| HIV | 42% (15/36) |  | 25% (21/85) | 39% (44/113) | 0.035 |
| History of HIV-PEP | 8% (3/36) |  | 11% (9/85) | 12% (14/114) | 0.712 |
| History of syphilis | 50% (18/36) |  | 39% (33/85) | 47% (54/114) | 0.229 |
| **Leading Symptom** |  |  |  |  |  |
| Asymptomatic | 81% (29/36) |  | 59% (50/85) | 75% (85/114) | <0.001 |
| Urethritis | 11% (4/36) |  | 34% (29/85) | 12% (14/114) |  |
| Proctitis | 6% (2/36) |  | 2% (2/85) | 11% (13/114) |  |
| Pharyngitis | 0% (0/36) |  | 1% (1/85) | 0% (0/114) |  |
| Cervicitis | 3% (1/36) |  | 4% (3/85) | 2% (2/114) |  |
| **Site of manifestation** |  |  |  |  |  |
| Urethral | 44% (16/36) |  | 76% (65/85) | 39% (45/114) | <0.001 |
| Anal | 47% (17/36) |  | 20% (17/85) | 55% (63/114) |  |
| Pharyngeal | 3% (1/36) |  | 1% (1/85) | 3% (3/114) |  |
| Cervical | 6% (2/36) |  | 2% (2/85) | 3% (3/114) |  |
| **Treatment** | 92% (33/36) |  | 88% (75/85) | 90% (103/114) | 0.631 |
| Doxycycline | 6% (2/33) |  | 16% (12/75) | 9% (9/103) | 0.142 |
| Negative follow-up test | N/A |  | 57% (4/7) | 100% (1/1) | 1 |
| Azithromycin | 94% (31/33) |  | 80% (60/75) | 31% (32/103) | <0.001 |
| Negative follow-up test | 90% (18/20) |  | 68% (26/38) | 90% (18/20) | 0.106 |
| Moxifloxacin | 0% (0/33) |  | 4% (3/75) | 60% (62/103) | <0.001 |
| Negative follow-up test | N/A |  | N/A | 85% (34/40) | N/A |
| **Concomitant infection** |  |  |  |  |  |
| Gonorrhea | 14% (5/36) |  | 13% (11/85) | 13% (15/114) | 0.163 |
| Chlamydia | 22% (8/36) |  | 22% (19/85) | 16% (18/114) | 0.239 |
| Syphilis | 14% (5/36) |  | 9% (8/85) | 18% (20/114) | 0.103 |

Abbreviations: HIV, human immunodeficiency virus; MSM, men who have sex with men; PEP, post-exposure prophylaxis, PrEP, pre-exposure prophylaxis.

**Table-S3. Patient characteristics per treatment outcome availability.**

|  | All episodes receiving treatment  N=178 | Treatment outcome not available  N=72 | Treatment outcome available  N=106 | p-value |
| --- | --- | --- | --- | --- |
| Age | 36.0 ± 10.6 | 34.4 ± 11.3 | 37.0 ± 10.0 | 0.053 |
| Male | 93% (165/178) | 88% (63/72) | 96% (102/106) | 0.028* |
| MSM | 20% (35/178) | 29% (21/72) | 13% (14/106) | 0.009* |
| On HIV-PrEP | 40% (72/178) | 28% (20/72) | 49% (52/106) | 0.005* |
| HIV | 32% (57/177) | 29% (21/72) | 34% (36/105) | 0.474 |
| History of HIV-PEP | 12% (22/178) | 14% (10/72) | 11% (12/106) | 0.609 |
| History of syphilis | 43% (77/178) | 38% (27/72) | 47% (50/106) | 0.201 |
| **Azithromycin resistance available** | 58% (103/178) | 58% (42/72) | 58% (61/106) | 0.917 |
| Azithromycin resistant episode | 68% (70/103) | 69% (29/42) | 67% (41/61) | 0.845 |
| **Leading Symptom** |  |  |  |  |
| Asymptomatic | 66% (118/178) | 65% (47/72) | 67% (71/106) | 0.143 |
| Urethritis | 22% (40/178) | 25% (18/72) | 21% (22/106) |  |
| Proctitis | 8% (14/178) | 4% (3/72) | 10% (11/106) |  |
| Pharyngitis | 1% (1/178) | 0% (0/72) | 1% (1/106) |  |
| Cervicitis | 3% (5/178) | 6% (4/72) | 1% (1/106) |  |
| **Site of manifestation** |  |  |  |  |
| Urethral | 53% (95/178) | 64% (46/72) | 46% (49/106) | 0.026* |
| Anal | 42% (74/178) | 32% (23/72) | 48% (51/106) |  |
| Pharyngeal | 2% (4/178) | 0% (0/72) | 4% (4/106) |  |
| Cervical | 3% (5/178) | 4% (3/72) | 2% (2/106) |  |

* We observed an increased proportion of available treatment outcomes for males, for MSM, for PrEP-users and for anal infections. This pattern suggests that the variables are dependent. To further investigate the underlying factors, we performed a binary logistic regression analysis with “outcome availability” being the dependent variable. Multivariate analysis enabled us to identified “PrEP-use” as the main contributor (aOR 2.14, 95%-CI 1.09-4.19) for treatment outcome availability. This finding seems plausible, since PrEP-users are usually connected to STI surveillance.

**Figure-S1:**


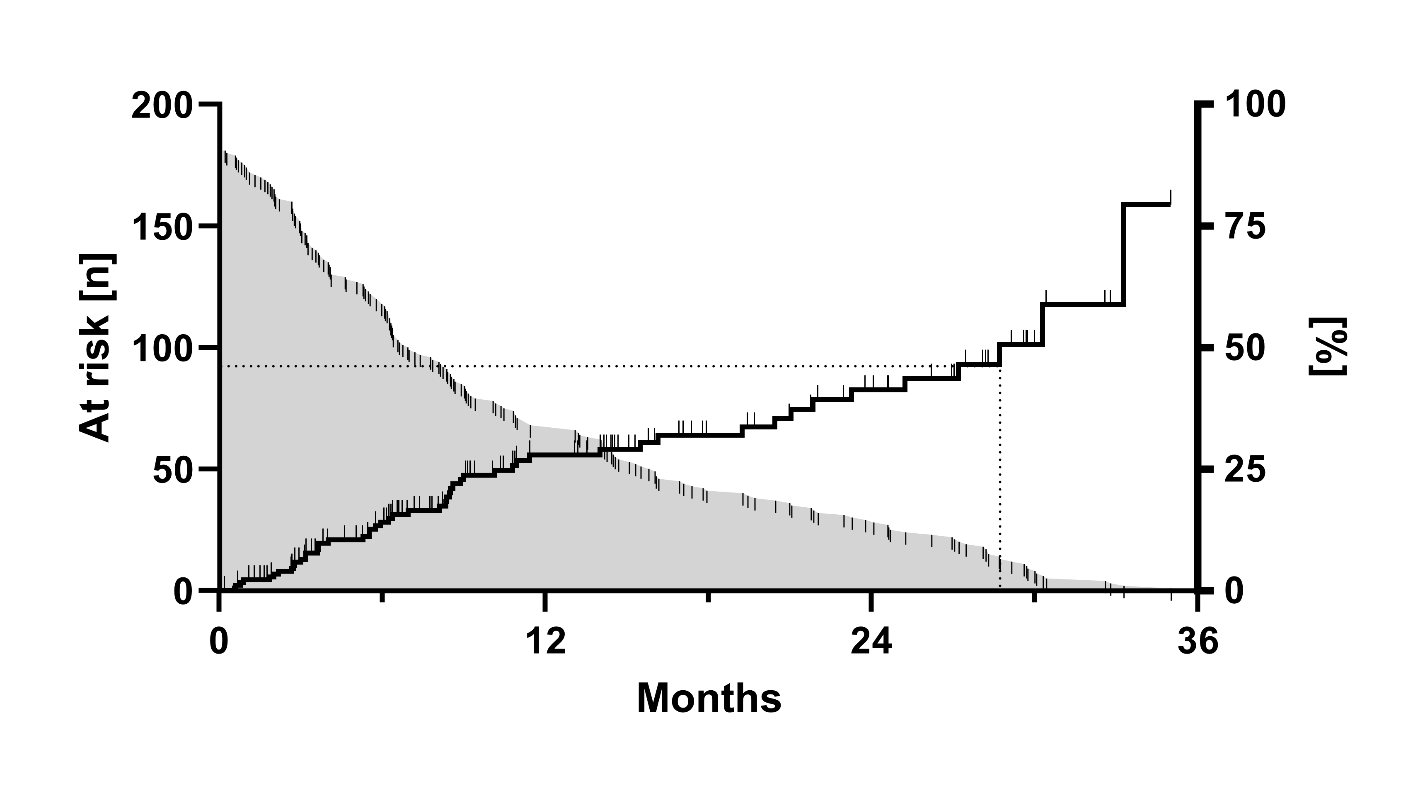


**Figure-S1. Subanalysis on PrEP-users: Kaplan-Meier-Curve describing the probability of the first *Mycoplasma genitalium* infection.**

Screening for sexually transmitted infections, including testing for *Mycoplasma genitalium*, every three months was performed for users of HIV pre-exposure prophylaxis. Thus, we had systematic follow-up data available for this specific population of interest and were able to perform a subgroup analysis: A survival analysis was used to calculate the median time from inclusion in the analysis (=first test) to first detection of *Mycoplasma genitalium*. Hundred-eighty-one PrEP-users with more than one available data point were analyzed, providing a median follow-up of 14.3 months. After 12 months, 28% had acquired a MG infection, whereas the median time until first positive MG test was 28.7 months. While the black line shows the longitudinal probability to acquire the first *Mycoplasma genitalium* infection, the grey curve indicates the declining number of individuals at risk.
